# Effect of Frying Process on the Flavor Variations of Allium Plants

**DOI:** 10.3390/foods12071371

**Published:** 2023-03-23

**Authors:** Jing Wang, Lina Qiao, Ruifang Wang, Ning Zhang, Yuping Liu, Haitao Chen, Jie Sun, Shuqi Wang, Yu Zhang

**Affiliations:** 1 Beijing Key Laboratory of Flavor Chemistry, Beijing Technology & Business University, Beijing 100048, China; 2 College of Food Science, Southwest University, Chongqing 400700, China

**Keywords:** Allium plants, frying process, flavor, aroma active compounds

## Abstract

The Allium plant is widely used in cuisines around the world for its characteristic flavor. The general profile of the plant changes a lot and presents quite different smells after the frying process. In this work, five Allium plants and their fried oils were compared to find out how the frying process impacts the general flavor profile. The results of sensory analysis indicated that the frying process could substantially increase the flavor acceptability of fresh Allium plants. Meanwhile, according to gas chromatography-mass spectrometry (GC-MS) analysis, fewer volatile compounds were detected in fresh Allium plants than in their fried oils. Furthermore, contents of nitrogen-containing compounds (ranging from 0.17 μg/g to 268.97 μg/g), aldehydes (ranging from 71.82 μg/g to 1164.84 μg/g), and lactones (ranging from 0 μg/g to 12.38 μg/g) increased significantly. In addition, more aroma-active substances were identified in the fried Allium oils revealed by gas chromatography-olfactometry (GC-O) analysis. Sulfur-containing compounds were the most abundant in fresh Allium plants, whereas nitrogen-containing compounds dominated in fried oils. The thermal degradation of sugars, amino acids and lipids as well as interactions between carbohydrates, proteins, and fats during the frying process were thought to be the main contributors to these variations. Therefore, this research provides a theoretical basis for the quality control of onion oil flavor and promotes the further development of the onion plant industry. Consequently, the research provided a theoretical basis for the quality control of Allium oils’ flavor and promoted the further development of Allium plant industries.

## 1. Introduction

Allium (*Allium* L.) is distributed in the northern hemisphere, with over 700 species [1]. In many countries, the Allium plant is one of the indispensable food ingredients. The most common Allium plants include garlic (*Allium sativum* L.), green onion (*Allium fistulosum* L.), leek (*Allium* tuberosu mrottl. ex spreng.), onion (*Allium cepa* L.), chive (*Allium schoenoprasum* L.), etc. Green onion, which is rich in sugars, proteins, amino acids, as well as some other nutrients [2] is one of the most common seasonings in Chinese cuisine. Garlic is a well-known edible and medicinal plant. Allicin in garlic is regarded to have healthcare functions, such as antiseptic, antibacterial, anti-cancer and anti-aging [3]. With a slightly spicy odor, shallot can be used for flavoring or deodorizing and can also increase appetite. Furthermore, onion, which is widely cultivated throughout the world, is known as the “queen of vegetables” with high nutritional values [4]. Additionally, bunching onion is frequently found in Thai and Cantonese cuisines. What’s more, consuming bunching onion can block the growth of cervical and skin cancer cells [5]. Accordingly, the five Allium plants are representative of high dietary and medical values. In general, a frying process can not only weaken the raw green notes of fresh Allium plants but also enhance the salty and roasted aroma characteristics [6,7]. Consequently, it is particularly significant to identify and compare the key aromatic compounds in Allium plants of different kinds before and after the frying process.

Volatile compounds of raw, roasted and fried Allium plants have been researched, and sulfides, disulfides, trisulfides and thiophenes were considered as the main components [8]. Some explorations using different heating methods found that the main volatile substances were 2-acrolein, 2,4-heptadienal, 3-methyl-2-butenal, dimethyl trisulfide, and 2,4-dimethylthiophene [9]. Additionally, sulfur-containing compounds and nitrogen-containing compounds were identified as the main factors affecting the whole flavor. For instance, dimethyl trisulfide, 2,4-dimethylthiophene, 2,5-dimethylpyrazine and 2-ethyl-3,6-dimethylpyrazine were the dominant compounds [10]. Besides, it was also found that volatile compounds in garlic were related to the heating methods. The flavor profiles of fried and roasted garlic were highly correlated with thioether and pyrazine compounds, specifically 2,6-dimethylpyrazine, dimethyl trisulfide, and diallyl disulfide [11]. Among them, the oil treatment of garlic favored the formation of vinyl disulfide [12].

Although some researches on the volatiles of fresh Allium plants have been reported, the flavor variations of Allium plants before and after the frying process is still lacking. Fresh Allium plants and their fried oils were chosen for our exploration because of the wide application of Allium plants and the great contribution of fried Allium oil to the flavor of Chinese cuisine, as well as its reflection of the cultural connotations of traditional Chinese dishes. Thus, in this work, representative Allium plants of different kinds, i.e., green onion, garlic, shallot, onion and bunching onion, were selected to analyze their characteristic flavor. Also, the major aroma-active compounds in fresh Allium plants and their fried oils were investigated through sensory analysis, gas chromatography-mass spectrometry (GC-MS) analysis, gas chromatography-olfactometry (GC-O) analysis and chemometrics method. The results will reveal how the frying process impacts the flavor characteristic of the Allium plants.

## 2. Materials and Methods

### 2.1. Materials and Chemicals

Green onion (*Allium fistulosm* L.), garlic (*Allium sativum* L.), shallot (*Allium ascaloncum* L.) and onion (*Allium cepa* L.) were from Beijing, and bunching onion (*Allium cepa* L. var. proliferum regel) was from Guangdong. The vegetable oil was soybean oil (Yuanbao). These were all purchased from local supermarkets in Beijing, and processed immediately after being brought to the laboratory. The solvent, dichloromethane, was from Thermo Fisher Scientific Inc. (Shanghai, China), and the internal standard, 1,2-dichlorobenzene, was from Aladdin Industrial Corporation (Shanghai, China). All of them were of HPLC grade. A mixture of alkanes from hexane (analytical reagent grade, ≥95% purity) to triacontane (Sigma-Aldrich Co., St. Louis, MI, USA) was used to assist in measuring the retention index (RI). Sodium chloride (AR) was from Sinopharm Chemical Reagent Co., Ltd. (Beijing, China).

### 2.2. Oil Preparation Process

The pretreatment methods for the five Allium plants’ ingredients were as follows [10]. Green onions with the white part were cut into 1 cm pieces, garlic was cut into 2 mm pieces and onions were cut into 1 cm pieces. Shallots were treated the same way as onions and bunching onions were treated the same way as garlic. A stainless-steel basin with a diameter of about 18 cm was placed on an induction cooker for frying. The temperature was measured with an oil thermometer during the frying process. For the green onions, for example, 300 g of soybean oil was added into the basin and heated to 140 °C. Then 300 g of fresh green onions were added to the hot oil for frying until the oil temperature reached 165 °C. After the oil temperature reached 165 °C, the fried green onion samples were separated immediately from the oil, and the oil was allowed to cool to room temperature as soon as possible. There is an unavoidable temperature change in this process, that is, the oil temperature will plummet to about 100 °C after adding fresh green onion plants, and then gradually increase. The mixture was continuously stirred during this process to prevent uneven heating. The above process was repeated twice with new oil every time and mixed the oil for subsequent analysis. The different kinds of fresh samples were prepared in the same way. Their fried oils were named green onion-oil, garlic-oil, shallot-oil, onion-oil and bunching onion-oil, respectively.

### 2.3. Sensory Analysis

Sensory analysis was conducted by a panel of ten members (six females and four males, aged 23 to 30). They were trained in quantitative descriptive analysis (QDA) and experienced in the sensory analysis of food samples [13]. The analysis was carried out in a sensory evaluation room at room temperature and relative humidity. The sensory analysis results can be used not only to compare the flavor differences of the samples, but also to assess the consistency of the instrumental analysis.

Samples (10 g) of fresh Allium plants and their fried oils were placed in covered, odorless, transparent polyethylene terephthalate bottles (volume = 30 mL) separately and randomly arranged. Initially, ten sensory panelists were asked to complete a preliminary round of descriptive sensory evaluation. This round of evaluation did not include any descriptors that might influence their decision. Participants were instructed to write down descriptors that may be used to characterize the flavor characteristics they actually perceived in the samples. Following that, all of the descriptors were iteratively debated and re-evaluated to determine whether or not to maintain them. As a result, the organoleptic descriptors for fresh Allium plants adopted by the members were fresh onion-like, fresh garlic-like, green grass, pungent, sweet, and acidic. Additionally, descriptors obtained for the Allium oils included onion-like, garlic-like, salty, fried, burnt, bitter, cooked vegetable, pungent, green grass, and oily. Subsequently, the samples were presented to the evaluators with the letter codes to recognize the aforementioned organoleptic descriptions and rate their scent intensities ranging from 0 (none) to 9 (very strong). The final score for each odor attribute was the average of the ratings of each panelist, and the obtained data were analyzed by principal component analysis (PCA).

### 2.4. Isolation of the Volatile Compounds

#### 2.4.1. Direct Solvent Extraction (DSE) of Fresh Allium Plants

The volatile compounds of fresh Allium plants were extracted by direct solvent extraction at room temperature (20 ± 5 °C). 20 g of fresh Allium samples were chopped and put into an Erlenmeyer flask, 100 mL of distilled water was added into the flask, and then analytical-grade sodium chloride was added to saturate the solution. The above mixture was homogenized at 1500 rpm for 5 min using a SCILO-GEX BlueSpin LED digital hotplate magnetic stirrer (MS-H280-Pro, Berlin, CT, USA). An internal standard solution (1000 μL, 26.0 μg/mL of 1,2-dichlorobenzene) and dichloromethane were added into the mixture for extraction. The mixtures were stirred at 1500 rpm for 30 min at room temperature during extraction. The solvent phase was separated by centrifugation at 3000 rpm and the mixture was centrifuged at 4 °C for 10 min. The procedure was repeated twice and the resulting solvent extract was used for solvent-assisted flavor evaporation (SAFE). The above steps were repeated for each fresh Allium plant.

#### 2.4.2. Solvent-Assisted Flavor Evaporation (SAFE)

50 g of fried oil sample was dissolved in 200 mL of dichloromethane. In addition, 1,2-dichlorobenzene (50 μL, 0.236 μg/μL) was added as an internal standard before the following procedure. The solution was distilled in a high vacuum using SAFE, a method that removes non-volatile materials [14,15,16]. The SAFE distillate was dried over anhydrous Na_2_SO_4_ and then filtered, and the solution was concentrated to about 6 mL using a Vigreux column (50 cm × 1 cm). The extracts were concentrated to 0.5 mL using a gentle stream of nitrogen gas. Samples were prepared in triplicate and stored in 2 mL glass vials at −40 °C for instrumental analysis. Each fresh Allium and their fried oil were prepared the same way.

### 2.5. Gas Chromatography-Mass Spectrometry Analysis

The GC-MS analysis was performed using an ISQ LT-TRACE 1310 (Thermo Fisher Scientific, Waltham, MA, USA) coupled with a Thermo Fisher mass spectrometer (Thermo Fisher Scientific, Waltham, MA, USA). GCMS-QP2010 (Shimadzu, Japan) was also used. Separations were performed on a TG-WAX column (30 m × 0.25 mm × 0.25 μm, Thermo Fisher Scientific, Waltham, MA, USA). Helium was used as the carrier gas and delivered to the column at a fixed flow rate of 1.0 mL/min. The GC oven temperature was initially 40 °C, followed by a 1 min hold, ramped at 4 °C/min to 220 °C, and held for 8 min. The mass detector conditions were as follows. EI ionization source, ionization energy, 70 eV, ion source temperature, 250 °C, transfer line, 250 °C, mass range, m/z 35–50 amu, injection volume, 1 μL, injector temperature, 250 °C, the sample was injected in a 1:5 split ratio and the mass range was operated with a solvent delay of 6 min for the samples.

### 2.6. Gas Chromatography-Olfactometry Analysis

GC-O analysis was carried out using a trace GC-MS system equipped with an olfactometer detector (ODP3, Gerstel, Mülheim an der Ruhr, Germany). TG-WAX was used as the analytical column, and the other GC parameters were identical to those of GC-MS analysis. Samples were sniffed by three trained and experienced assessors (two females and one male) and retention times (RT) of elution peaks and odor descriptions were recorded during the sniffing process.

### 2.7. Identification and Quantification of the Aroma Compounds

The volatile compounds in fresh Allium plants and fried Allium plants oils were initially identified by comparing their mass spectra with those of the NIST 2014 database. The homologous series of standards of n-alkanes (C6~C30) were analyzed under the same chromatographic conditions. The retention index (RI) of the detected compounds was calculated and compared with the RI in the NIST 2014 database using the same capillary column. The results were used in the equation [17] to calculate the RI of each compound.

The relative concentrations of the aromatic compounds were calculated by correlating the volatiles’ peak areas with the internal standard’s peak. The amount of each compound can be calculated using the equation [18].

### 2.8. Statistical Analysis

Results are expressed as the mean ± standard deviation (SD) of at least three independent pretreatment experiments (extraction procedure) for different fresh Allium plants and fried oils. Principal component analysis (PCA, carried out by XLSTAT v. 2018) was performed to differentiate the samples. The Venn was conducted by jvenn (jvenn: an interactive Venn diagram viewer (inra.fr)). The circular plot and the heat map were drawn by Origin 2022 software.

## 3. Results and Discussion

### 3.1. Sensory Analysis

PCA analysis was performed based on the sensory analysis results of different kinds of fresh Allium plants and their fried oils. The data were pre-processed before analysis. The mean values of sensory analysis were used for analysis and the outliers were removed.

**Figure 1:**
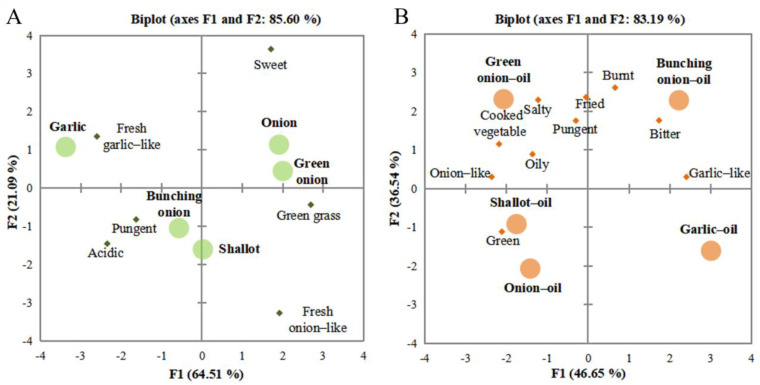
Principal component analysis for sensory analysis of the five samples. ((**A**) fresh Allium plants, (**B**) fried Allium oils). Note: the light green circular symbols indicate five fresh Allium plants; the green diamond signs indicate the sensory evaluation descriptions. The light orange circular symbols indicate five fried Allium oils; the orange diamond signs indicate the sensory evaluation descriptions. Fresh Allium plants: green onion, garlic, onion, shallot, and bunching onion. Fried Allium oils: green onion-oil, garlic-oil, onion-oil, shallot-oil and bunching onion-oil.

The PCA biplot of the five fresh Allium plants was shown in Figure 1A. The first principal component (PC1) and second principal component (PC2) accounted for 64.51% and 21.09% of the variance, respectively. These two components together explained 85.60% of the total variance. The variance contribution of PC1 was more outstanding than that of PC2, indicating that the greater the distance of PC1 axis, the greater the sample variation. According to the result, all fresh Allium plant samples were separated on the PCA plot, indicating that the samples differed somewhat in volatile composition. Using the center line as the dividing line, it was obvious that there were significant aromatic differences among the samples. In the plot, onion and green onion were on the right side, and garlic and bunching onion were on the left side. Onion and green onion mainly produced a sweet note. The smell of fresh garlic was mainly fresh garlic-like although the note of bunching onion was primarily strong pungent and acidic.

The PCA biplot of the five Allium oils is shown in Figure 1B. The two principal components (PC1 and PC2) accounted for 83.19% of the variance (46.65% and 36.54%, respectively), indicating that the two principal components can reflect the basic information of the original data of the samples. According to the PCA results, sensory attributes, including salty, fried, cooked vegetable, oily, pungent and onion-like, were more related to the characteristics of green onion-oil. Shallot-oil and onion-oil exhibited a relatively high tendency with the characteristics of green grass. Also, bunching onion-oil revealed the characteristics of burnt, bitter, and garlic-like. The flavor profile of garlic-oil showed a more significant difference from the other samples.

Therefore, the thermal treatment process greatly impacted the flavor profile of these fresh Allium plants. This process enhanced the overall profile, which also produced unique odorants. This phenomenon was due to the fact that many chemical reactions occurred during this frying process, such as interactions and thermal degradations between Allium plants and matrix under high-temperature conditions [19]. Furthermore, a large number of aroma substances were generated during this process, leading to the notes of salty, fried, oily, cooked vegetable, and other similar aromas. Thus, the frying process can substantially increase the flavor acceptability of fresh Allium plants.

### 3.2. Flavor of Fresh Allium Plants

#### 3.2.1. GC-MS Analysis

A detailed analysis of their aroma substances was carried out to investigate further the factors affecting the flavor profiles of fresh Allium plants. GC-MS analysis was applied to analyze the volatile compounds in the five fresh Allium plant samples, and the results are shown in Table A3. In general, a total of 69 volatile compounds were identified in five fresh Allium plants, including 40 sulfur-containing compounds, 6 alcohols, 7 ketones, 6 aldehydes, 4 acids, 3 esters, 2 heterocyclic compounds and 1 nitrogen-containing compound. Each fresh Allium plant had a similar number of volatile components. Green onion had 29 volatile components, garlic had 28 volatile components, shallot had 27 volatile components, onion had 21 volatile components, and bunching onion had 32 volatile components. The extracts of all five fresh Allium plants exhibited a rich aromatic profile.

In terms of the content of detected components, the highest content of volatile compounds was sulfur-containing compounds (1860.42 μg/g). Besides, contents of ketones (76.98 μg/g), alcohols (47.76 μg/g), aldehydes (71.82 μg/g), and acids (52.48 μg/g) were relatively similar, whereas heterocyclic compounds and nitrogen-containing compounds were the least similar. The concentrations of sulfur-containing compounds in bunching onion, green onion, shallot, garlic and onion ranged from 182.08 µg/g to 599.67 µg/g, indicating that sulfur-containing compounds play a significant role in fresh Allium plants’ flavor. Compounds, hexanal and octanoic acid, were detected in all five fresh Allium plants. Their contents did not differ enormously, indicating that they did not play a dominant role in the overall flavor.

#### 3.2.2. GC-O Analysis

**Figure 2:**
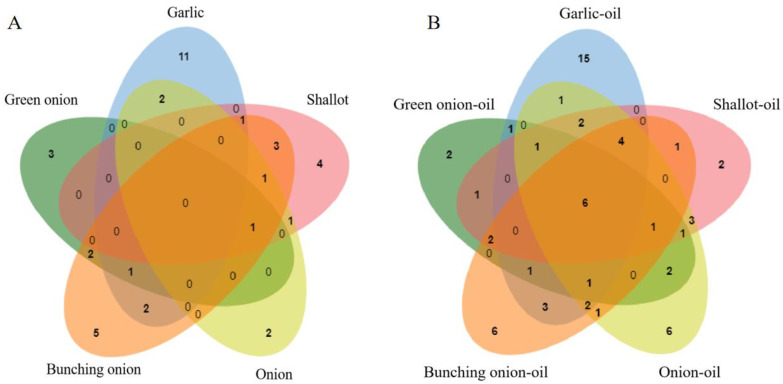
The Venn diagram of aroma substances identified in gas chromatography- olfactometry (GC-O) analysis in fresh Allium plants (**A**) and fried Allium oils (**B**). Note: the numbers in the graph indicate the number of compounds.

The aroma-active substances in fresh Allium plants were characterized by GC-O analysis. It showed that 42 volatile substances were characterized as odor-active compounds, including 2 alcohols, 4 aldehydes, 1 ketone, 1 phenolic, 1 acid, 1 ester, 2 heterocyclics, 26 sulfur-containing compounds, 1 nitrogen-containing compound and 3 unknown compounds (Table A1). As shown in Figure 2A, the Venn figure was plotted based on the identified volatile compounds in different fresh Allium plants. Accordingly, three, eleven, four, two, and five specific volatile compounds were only identified in green onion, garlic, shallot, onion and bunching onion, respectively. It was worth noting that vanillin and furaneol presented only aroma-active qualities in green onion in this work. These two compounds present sweet and creamy aromas, broadly used in the food industry. Additionally, some sulfur-containing compounds, such as diallyl sulfide, methyl allyl disulfide, dimethyl trisulfide and diallyl trisulfide, only presented aroma characteristics in garlic [20]. These compounds were the main elements in the profile of garlic. Odorants were identified in shallot, including propyl sulfide and 2-methoxy-3-(1-methyl-propyl) pyrazine. And nonanoic acid and prenylthiol were only detected in onion. The compounds identified only in bunching onion were dimethyl disulfide, 3-methylthiophene and 2-hexyl-5-methyl-3(2H)-furanone. These results have hardly been researched before, and are rarely reported. In addition, bunching onion and shallot have three compounds in common. 2-Methyl-2-pentenal (green), propyl disulfide (alliaceous), 3-mercapto-1-hexanol (sulfurous); this is in line with the results of sensory analysis. That is to say, these compounds are critical elements in the formation of pungent and sour aromas in sensory analysis results.

#### 3.2.3. Sulfur-Containing Compounds

Generally, threshold values of sulfur-containing compounds (44 μg/kg) [21] are the lowest of all types of aroma-active substances. Usually, the contents of sulfur-containing components are pretty low, but they significantly impact the overall profile. Thus, they play a critical role in the overall flavor profile of foods. Sulfur-containing compounds have a pungent aroma. However, when diluted to concentrations of ppb or ppm, they can present aromas of fresh onions and garlic [22]. Combined with the analytical results, it can be observed that sulfur-containing compounds dominated the identified aroma active compounds of fresh Allium plants. A total of 26 sulfur-containing compounds were detected to possess aroma-active characteristics in the fresh Allium plants, which can be broadly classified into monosulfide, disulfide, trisulfide, etc. Among them, the number of trisulfides was relatively higher; they were dimethyl trisulfide, methyl propyl trisulfide, methyl allyl trisulfide, diallyl trisulfide, etc. Similarities can be found between the identified compound species compared to existing researches [23]. Moreover, the differences between different Allium plants may largely stem from the origin and growth environment of the raw materials. As for the concentration, propyl disulfide, which presented an alliaceous note, was the most highly concentrated compound among these fresh Allium plants, at a concentration of 304.01 μg/g. Other compounds with high content were dipropyl trisulfide (155.38 μg/g, garlic) and dimethyl trisulfide (146.58 μg/g, cooked onion). They have been considered key odorant compounds in fresh Allium plants. In addition, thiophene was detected in all fresh samples, which is responsible for the roast flavor. The most abundant thiophene was 3,4-dimethylthiophene at 102.14 μg/g in fresh plants.

### 3.3. Flavor of Fried Allium Oils

#### 3.3.1. GC-MS Analysis

In order to explore how the frying process affected the flavor profile of the fresh Allium plants, the aroma components of their fried oils were carefully conducted. Results of volatile compounds in the five fried Allium oils via GC-MS are shown in Table A4. A total of 150 volatile compounds were identified, including 19 alcohols, 23 aldehydes, 23 ketones, 9 acids, 4 esters, 2 lactones, 28 sulfur-containing compounds, 29 nitrogen-containing compounds, and 13 heterocyclic compounds. Among the five fried Allium oils, sulfur-containing compounds and nitrogen-containing compounds were the most abundant, followed by esters and lactones. For the identified volatiles by GC-MS analysis, aldehydes had the highest content, followed by sulfur-containing compounds and heterocyclics. Besides, alcohols, ketones, acids, and nitrogen-containing compounds were moderately abundant in fried Allium oils. Aldehydes were the most abundant; they presented the highest content in onion-oil (433.6 μg/g) and shallot-oil (312.81 μg/g), but less content in garlic-oil (15.58 μg/g). The same phenomenon could be observed in the results of the sensory analysis. The flavor profiles of onion-oil and shallot-oil were closer, although garlic-oil differed the most from the other Allium oils. The closer kinship between shallot and onion may be the reason for their similar aroma [24]. The large morphological differences between garlic and other Allium plants may be the reason for the significant distinction in aroma between garlic-oil and other fried Allium oils. Moreover, the difference in the quantity of aroma substances between different fried Allium oils was not apparent. The common compounds of the fried Allium oils were mainly aldehydes and sulfur-containing compounds, such as (*E*, *E*)-2,4-heptadienal, (*E*, *E*)-2,4-decadienal, dimethyl disulfide, and dimethyl trisulfide.

#### 3.3.2. GC-O Analysis

Aroma-active compounds in fried Allium oils were characterized by GC-O analysis. A total of 68 aroma-active compounds were detected, including 7 alcohols, 16 aldehydes, 6 ketones, 3 acids, 1 lactone, 9 heterocyclic compounds, 12 sulfur-containing compounds, 10 nitrogen-containing compounds and 4 unknown compounds (Table A2). As revealed by Figure 2B, 2, 15, 2, 6, and 6, specific volatile compounds only existed in green onion-oil, garlic-oil, shallot-oil, onion-oil and bunching onion-oil, respectively. Accordingly, dihydropyranone was only identified as odorant in green onion-oil. Methyl allyl disulfide, diallyl trisulfide, ethenylpyrazine and 2-methyl-5-propenylpyrazine were simply considered as aroma components in garlic-oil. The compounds identified only in shallot-oil were 2-hexenal. In onion-oil, odorants including (*Z*)-2-pentenol were identified. As well as compounds of thiophene, (*E*)-1-propenyl propyl trisulfide and 2,6-dimethylpyrazine were only detected in bunching onion-oil. These compounds have been reported less in previous researches. There were six compounds, 1-octen-3-ol (mushroom and earthy, aroma intensity = 4), (*E*)-2-octenal (cucumber and herbal, 2), (*E*, *E*)-2,4-decadienal (oily and meat, 3), dimethyl trisulfide (sulfurous and cooked onion, 3), 2-furyl methyl ketone (sweet, 3) and furaneol (cotton and caramel, 4), that presented in all fried Allium oils. These compounds have high aroma intensity, and were perceived as the main odorants of fried Allium oils. Furthermore, (*E*, *E*)-2,4-decadienal presented mainly oily notes and were speculated to contribute to the organoleptic descriptors of oily, salty and fried. Some sulfur-containing compounds, such as dimethyl trisulfide, presented sulfurous and cooked onion aromas and were deemed to contribute to the strong onion-like, garlic-like, and pungent notes of fried Allium oils, as shown in the sensory evaluation results. Besides, (*E*)-2-octenal displayed cucumber and herbal notes, related to cooked vegetable and green descriptors. Other aroma substances enriched the overall aroma of fried Allium oils.

### 3.4. Comparison of Fresh Allium Plants and Their Fried Oils

#### 3.4.1. Comparison of GC-MS Analysis Results

**Figure 3:**
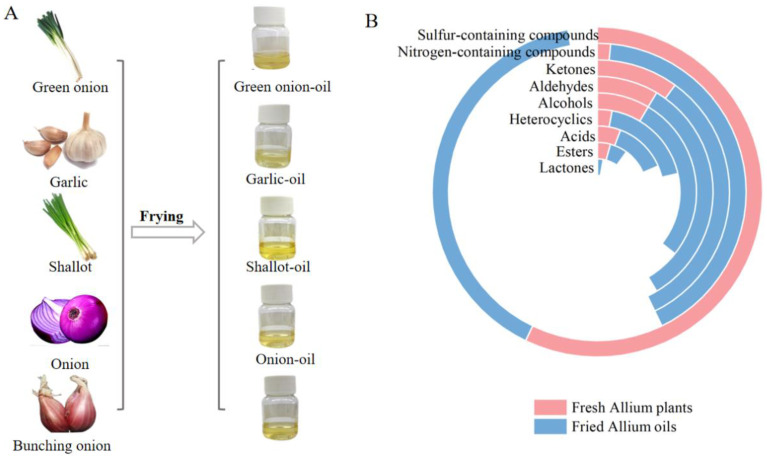
A comparative circular plot of the number of volatile compounds in fresh Allium plants and their fried oils. (**A**) The fresh Allium plants and their fried oils. (**B**) A comparative circular plot. Specific quantities are detailed in Table A5.

A total of 69 volatile compounds were identified in five fresh Allium plants, and 150 were found in their fried oils. Compared with fresh Allium plants, there was a sharp increase in the amounts of volatile compounds in their fried Allium oils, demonstrating that the frying process significantly affected the flavor profiles. As shown in Figure 3, more sulfur-containing compounds were in the fresh Allium plants than in the fried ones. Conversely, the other types of compounds in fresh Allium plants, such as nitrogen-containing compounds, ketones, aldehydes, alcohols, heterocycles, acids, esters, and lactones, were less than those in the fried oils. In particular, only one nitrogen-containing compound was detected in fresh plants, however, this quantity increased to 29 in their fried oils. In addition, lactones were only presented in the fried Allium oils rather than the fresh ones, which were produced after the frying process and gave creamy and sweet aromas in order to make the overall flavor more layered.

Meanwhile, it was worth noting that the contents of sulfur-containing compounds dropped significantly after the frying process from 1860.42 μg/g to 468.94 μg/g, whereas nitrogen-containing compounds and aldehydes increased considerably. The content of nitrogen-containing compounds in fresh Allium plants was 0.17 μg/g, which grew to 268.97 μg/g in their fried oils. Simultaneously, aldehydes were detected in fresh Allium plants at a concentration of 71.82 μg/g and in their fried oils at 1164.84 μg/g. The reaction between lipids, sugars and volatile precursors in Allium plants at high temperatures may be a possible reason for the above content changes. Generally, propyl disulfide might play an essential role in the flavor profile of Allium plants due to its low odor threshold value [25]. It was detected in fresh green onion at a maximum concentration of 192.32 μg/g. (*E*, *E*)-2,4-decadienal contributed a desirable fried aroma to the fried Allium oils in small amounts, but provided an oily and rancid flavor in high amounts [26]. In this work, (*E*, *E*)-2,4-decadienal was detected in all 5 oils with levels not exceeding 83 μg/g. It contributed to the fried note, consequently. In addition, alkyl pyrazines were the primary type of nitrogen-containing compounds, related to the roasted and nutty aroma. They were once reported as a primary contributor to cooked, roasted, and fried foods [26,27]. A total of 13 alkyl pyrazines were identified in this work. Among them, 2,5-dimethylpyrazine was found to have the highest content (64.71 μg/g) and contributed more to the fried flavor.

**Figure 4:**
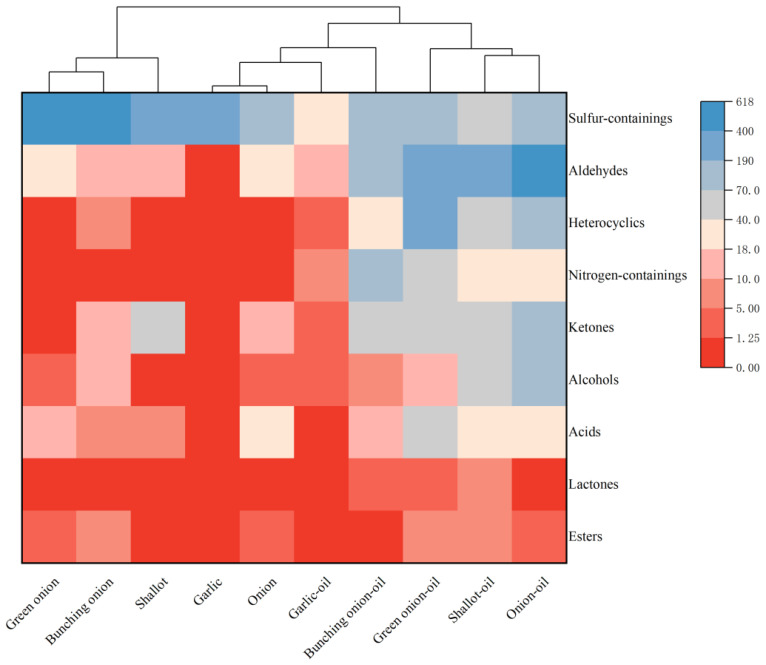
A heat map showed the volatile compound concentration present in five fresh Allium plants and their fried oils.

As an efficient tool for the evaluation of product quality, a heat map can visualize the trend of changes in the content of volatile compounds in tested samples [28]. A total of 9 types of compounds were compared and performed into a heat map, covering the identified components of the five fresh Allium plants and their fried oils (Figure 4). Affected by the frying process, there was a decrease in the number of sulfur-containing compounds, phenols, and an increase in the number of alcohols, aldehydes, ketones, acids, esters, lactones, nitrogen-containing compounds and heterocyclic compounds. Among them, aldehydes, nitrogen-containing compounds and heterocyclic compounds showed the greatest increase, more specifically, unsaturated aldehydes, and furans increased the most in number. Therefore, we can conclude that the most crucial contributors to the flavor of Allium plants after the frying process were unsaturated aldehydes, nitrogen-containing compounds and furans. It means that the frying process significantly changed the main flavor profile of the samples, and the process caused substantial sensory differences. Previous work had reported that the most critical flavor contributors of fried green onion and fried garlic were mainly sulfur-containing compounds, pyrazines, and unsaturated aldehydes [29]. This is consistent with the results of this research. In addition, the sulfur-containing compounds differed in type and content, such as the more significant flavor contribution of methyl sulfide in green onion-oil [8,9,11,30] and the predominant one in garlic-oil was allyl sulfide [12,31]. This is related to the difference in the sulfur-containing compounds in raw materials of fresh onions and garlic.

#### 3.4.2. Comparison of GC-O Analysis Results

A total of 16 compounds were identified in both fresh Allium plants and their fried oils via GC-O analysis. Specifically, these were five disulfides (dimethyl disulfide, diallyl disulfide, methyl allyl disulfide, methyl propyl disulfide, (*E*)-methyl-1-propenyl disulfide), five trisulfides (diallyl trisulfide, methyl propyl trisulfide, (*E*)-1-propenyl propyl trisulfide, methyl allyl trisulfide, dimethyl trisulfide), two unsaturated aldehydes (2-undecenal, 2-methyl-2-pentenal), two aldehydes (hexanal, 1-nonanal), one acid (nonanoic acid), and furaneol. It is easy to know that these compounds do not constitute newly generated unique flavors such as roasted aroma after frying. They mainly present the characteristic aroma profile of the Allium plants, such as onion and garlic aromas, as well as other aromas that contribute to the overall aroma profile of the Allium plants.

**Figure 5:**
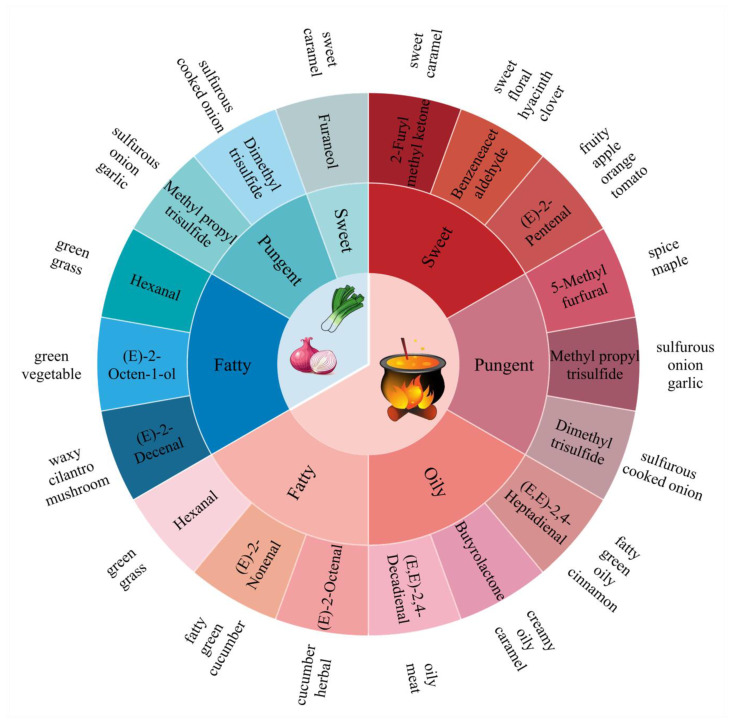
Flavor wheel of main odor characteristic components in five fresh Allium plants (in blue series) and their fried oils (in red series): component (third circle), subjective perception category (second circle) and related odor description (fourth circle).

In Figure 5, the blue series represented fresh Allium plants and the red series represented fried oils. The GC-O information of related compounds was sorted out, and the main volatile components (third circle) were identified in this study. It can be seen that there are similarities and differences in the aroma substances of fresh Allium plants and their fried oils. It could be observed that the fresh Allium plants primarily presented sweet, pungent, and fatty aromas. Meanwhile, the flavor profiles of fried Allium oils were mainly sweet, pungent, oily, and fatty notes. Among them, the same compounds, such as methyl propyl trisulfide, constituted a pungent aroma in fresh Allium plants and their fried oils. Furthermore, different aroma substances contributed the same flavor in the fresh plants and their oils, respectively. For instance, furaneol in fresh Allium plants contributed a sweet aroma profile, and 2-furyl methyl ketone, benzeneacetaldehyde, and (*E*)-2-pentenal in fried Allium oil embodied the sweet flavor profile.

#### 3.4.3. Analysis of Reasons for Change

Allium plants are rich in proteins and amino acids [32,33,34,35,36], and cooking oil is used as a heat-conducting medium in the frying process. Generally, for the fried Allium oils, 89 aroma-active substances were not present in fresh Allium plants. Those odorants were considered to be the productions of the interactions and thermal degradations between basic components. The basic components are the fats, proteins and carbohydrates in the system. The interactions between basic components referred to the interaction of carbohydrates, proteins and fats during heating. The Maillard reaction (browning reaction) is the most pivotal process between sugars and amino acids. Strecker aldehydes were often the primary Maillard reaction products during the first stage of the reaction because of the brief heating period and low temperature. These molecules can further combine to create scent compounds that are distinctive, such lactones and furans. Moreover, the Maillard reaction produced substances with baking aromas like pyrazines, pyrroles and pyridines. Combined with the identified compounds, furanol presented in all fried Allium oils. Furaneol is a critical aroma compound with sweet, bakery and caramel aromas [37], which is naturally present in fresh pineapple and beef broth. Nitrogen-containing compounds are also a class of compounds that increase significantly in content and type after the frying process, such as 2-methylpyrazine, 2-ethyl-6-methylpyrazine, and 3-ethyl-2,5-dimethylpyrazine. These compounds typically present a roasted and nutty aroma which also contributes considerably to the flavor profile of the oil. Thermal degradation reactions are divided into thermal degradation of sugars, fats and amino acids. It is known from other studies that the main product of the thermal degradation of sugar is 2-acetylfuran [10]. The sulfur-containing compounds that contributed more to the overall flavor of fried Allium oils were mainly produced by the thermal degradation reactions of amino acids, such as dimethyl trisulfide. Sulfur-containing compounds are characterized by a low threshold but high aroma intensity, and are the main components of meat aroma in Chinese cuisine [38]. Dimethyl trisulfide, which has a similar aroma to fresh onion and garlic, is naturally present in fresh onion with a threshold value of 0.005-0.01 ppb. It is produced through the degradation of sulfur-containing substances such as S-methyl-L-cysteine sulfoxide in onions [39]. Thermal degradation reactions of fats yield unsaturated aldehydes and methyl ketones, lactones and acids. These products are characteristic aromatic compounds of fried foods, which usually have fried and fatty notes. Among them, 2,4-decadienal is the characteristic aromatic compound of fried foods. In summary, the frying process enhances the salty and roasted aroma characteristics of the fresh Allium plants by not only weakening their original green notes.

## 4. Conclusions

Due to their characteristic flavor, Allium plants are frequently utilized in cuisines around the world, and the frying process can significantly impact their flavor profiles. This research aimed to explore the flavor changes between fresh Allium plants and their fried oils, as well as the possible causes of the changes. The results of sensory analysis presented significant flavor differences between fresh and fried samples. Fresh Allium plants mainly showed aromas of green, fresh garlic-like and fresh onion-like, whereas fried oils mainly showed fried, salty, fatty, burnt, garlic-like and onion-like notes. GC-MS analysis, GC-O analysis and statistical analysis revealed the flavor composition of fresh Allium plants and fried oils in more detail. Sulfur-containing compounds dominated the aroma-active compounds of the fresh Allium plants. In contrast, unsaturated aldehydes, nitrogen-containing compounds, and furans were the most critical contributors to the flavor of fried Allium oils. Furthermore, it was worth noting that the contents of sulfur-containing compounds dropped significantly after the frying process from 1860.42 μg/g to 468.94 μg/g, whereas nitrogen-containing compounds and aldehydes increased considerably. The reaction between lipids, sugars and volatile precursors in Allium plants at high temperatures may be a possible reason for the above changes. The subsequent study will focus on the contribution of each aroma substance to the overall flavor of the fried Allium oils. This will help to understand further and control the flavor stability of the fried Allium oil. Besides, product improvement and quality control can also be theoretically guided. In addition, the nutritional value, as well as the physicochemical properties of fried Allium oils, were also the focus of future research.

## Data Availability

Data available on request from the authors.

## Appendix A

**Table A1:** Comparative analysis of the aroma active compounds detected in five fresh Allium plants by gas chromatography-olfactometry (GC-O).

No.	Compounds ^a^	Odor Descriptor ^b^	Aroma Intensity					Identification ^c^
			Green Onion	Garlic	Shallot	Onion	Bunching Onion	
Alcohols								
1	2-Ethylhexanol	fruity	-	1	-	4	-	MS,RI,O
2	2-Hexyl-1-decanol	tar	-	-	3	-	-	MS,RI,O
Aldehydes								
1	Hexanal	fresh, green	-	1	1	-	1	MS,RI,O
2	2-Methyl-2-pentenal	green	-	-	2	-	1	MS,RI,O
3	1-Nonanal	waxy, orange	-	2	-	-	1	MS,RI,O
4	2-Undecenal	fruity	-	3	-	-	-	MS,RI,O
Ketones								
1	Acetoin	milky	-	-	1	-	-	MS,RI,O
Phenolics								
1	Vanillin	vanilla	2	-	-	-	-	MS,RI,O
Acids								
1	Nonanoic acid	creamy	-	-	-	2	-	MS,RI,O
Esters								
1	Methyl palmitate	oily	-	1	-	-	-	MS,RI,O
Sulfur-containing compounds								
1	3-Methyl-2-butenethiol	sulfurous	-	-	-	2	-	MS,RI,O
2	Dimethyl disulfide	vegetable	-	-	-	-	1	MS,RI,O
3	Allyl N-propyl sulfide	green onion	-	-	-	-	1	MS,RI,O
4	Propyl sulfide	garlic	-	-	1	-	-	MS,RI,O
5	Diallyl sulfide	garlic	-	2	-	-	-	MS,RI,O
6	Methyl propyl disulfide	onion	2	-	-	-	1	MS,RI,O
7	2-Methyl-3-furanthiol	fishy	-	2	-	-	-	MS,RI,O
8	Methyl allyl disulfide	garlic	-	3	-	-	-	MS,RI,O
9	Propyl disulfide	alliaceous	-	-	2	-	1	MS,RI,O
10	Dimethyl trisulfide	cooked onion	-	3	-	-	-	MS,RI,O
11	1-Heptanethiol	onion	1	-	-	-	-	MS,RI,O
12	Diallyl disulfide	nut	3	4	-	-	3	MS,RI,O
13	Methyl propyl trisulfide	onion	2	-	-	-	2	MS,RI,O
14	Methyl allyl trisulfide	garlic	-	3	-	-	-	MS,RI,O
15	Dipropyl trisulfide	garlic	-	-	4	3	2	MS,RI,O
16	3,5-Dimethyl-1,2,4-trithiolane	meaty	-	-	-	-	2	MS,RI,O
17	Diallyl trisulfide	garlic	-	4	-	-	-	MS,RI,O
18	3-Mercapto-1-hexanol	sulfurous	-	-	4	-	3	MS,RI,O
19	3-Methylthiophene	fatty	-	-	-	-	1	MS,RI,O
20	2-Methylthiophene	alliaceous	-	-	1	1	-	MS,RI,O
21	3,4-Dimethylthiophene	roasted onion	-	1	-	1	-	MS,RI,O
22	2-Ethylthiophene	garlic	-	2	-	-	-	MS,RI,O
23	(*E*)-Methyl-1-propenyl disulfide	sauce	-	2	-	-	2	MS,RI,O
24	1,2-Dithiacyclopentene	coconut	-	3	-	-	-	MS,RI,O
25	(*E*, *E*)-Propenyl disulfide	coconut	-	2	-	-	-	MS,RI,O
26	(*E*)-1-Propenyl propyl trisulfide	coconut	2	-	3	4	2	MS,RI,O
Nitrogen-containing compounds								
1	2-Methoxy-3-(1-methyl-propyl) pyrazine	caramel	-	-	4	-	-	MS,RI,O
Heterocyclics								
1	Furaneol	sweet	3	-	-	-	-	MS,RI,O
2	2-Hexyl-5-methyl-3(2H)-furanone	sweet	-	-	-	-	1	MS,RI,O
Unknown								
1	unknown	almond	-	-	4	-	-	O
2	unknown	paint	-	4	-	-	-	O
3	unknown	plum	-	4	-	-	-	O

^a^ Aroma compounds detected in fresh Allium plants. ^b^ Odor description perceived by GC−O analysis. ^c^ Identification based on Nist 14 mass spectral database (MS); published RIs; published odor descriptions (O). ‘-’ not detected.

**Table A2:** Comparative analysis of the aroma compounds detected in five fried Allium oils by GC-O.

No.	Compounds ^a^	Odor Descriptor ^b^	Aroma Intensity					Identification ^c^
			Green Onion-Oil	Garlic -Oil	Shallot-Oil	Onion -Oil	Bunching Onion-Oil	
Alcohols								
1	Ethyl vinyl carbinol	fruity	-	-	1	-	-	MS,RI,O
2	1-Pentanol	sweet	-	2	-	-	-	MS,RI,O
3	(*Z*)-2-Pentenol	spicy	-	-	-	4	-	MS,RI,O
4	1-Octen-3-ol	mushroom	4	4	3	4	4	MS,RI,O
5	(*E*)-2-octenol	fruity	-	1	2	2	-	MS,RI,O
6	1-(2-Butoxyethoxy)ethanol	milky	-	-	-	1	-	MS,RI,O
7	1-Hexadecanol	waxy	-	2	-	-	-	MS,RI,O
Aldehydes								
1	(*E*)-Crotonaldehyde	pungent	-	-	-	-	1	MS,RI,O
2	Hexanal	green grass	-	2	1	1	1	MS,RI,O
3	(*E*)-2-Pentenal	fruity	-	1	3	2	2	MS,RI,O
4	2-Methyl-2-pentenal	Fruity, grassy	-	-	-	-	4	MS,RI,O
5	Heptaldehyde	Green, aldehydic	-	-	-	2	-	MS,RI,O
6	2-Hexenal	fruity, green	-	-	1	-	-	MS,RI,O
7	1-Nonanal	waxy, citrus	-	-	-	2	-	MS,RI,O
8	(*E*)-2-Octenal	green, citrus	2	2	1	2	1	MS,RI,O
9	(*E*, *E*)-2,4-Heptadienal	fatty green	-	2	1	3	2	MS,RI,O
10	(*E*)-2-Nonenal	soapy	2	-	1	2	-	MS,RI,O
11	p-Tolualdehyde	fruity, cherry	-	1	-	-	-	MS,RI,O
12	Benzeneacetaldehyde	floral, rose	4	-	3	-	3	MS,RI,O
13	(*E*)-2-Decenal	fatty, aldehydic	-	4	3	3	-	MS,RI,O
14	(*E*, *E*)-2,4-Nonadienal	fatty, chicken	3	-	-	2	-	MS,RI,O
15	(*E*, *E*)-2,4-Decadienal	oily, meat	3	4	4	3	1	MS,RI,O
16	2-Undecenal	nut	-	4	-	-	-	MS,RI,O
Ketones								
1	Ethyl vinyl ketone	peppery	-	-	3	1	-	MS,RI,O
2	Hydroxy-3-methyl-2-butanone	vanilla	-	2	-	-	-	MS,RI,O
3	2,3-Octadione	cilantro	-	2	-	2	-	MS,RI,O
4	3-Nonen-2-one	phenolic	-	3	-	-	-	MS,RI,O
5	4-Cyclopentene-1,3-dione	green	2	-	2	-	-	MS,RI,O
6	Methyl cyclopentenolone	caramel	-	-	-	1	-	MS,RI,O
Acids								
1	Hexanoic acid	sweat	-	-	2	2	-	MS,RI,O
2	Octanoic acid	rancid	3	-	-	-	-	MS,RI,O
3	Nonanoic acid	waxy, creamy	-	-	-	-	2	MS,RI,O
Lactones								
1	Butyrolactone	creamy	3	-	3	-	3	MS,RI,O
Sulfur-containings								
1	Dimethyl disulfide	vegetable	2	1	-	-	-	MS,RI,O
2	Methyl propyl disulfide	onion	-	-	-	1	2	MS,RI,O
3	Methyl allyl disulfide	garlic	-	3	-	-	-	MS,RI,O
4	Dimethyl trisulfide	cooked onion	3	4	3	4	3	MS,RI,O
5	Allyl isothiocyanate	mustard	-	2	-	-	-	MS,RI,O
6	Diallyl disulfide	nut	-	4	-	-	-	MS,RI,O
7	Methyl propyl trisulfide	onion	2	-	2	3	3	MS,RI,O
8	Methyl allyl trisulfide	garlic	-	4	-	-	-	MS,RI,O
9	Diallyl trisulfide	garlic	-	3	-	-	-	MS,RI,O
10	Thiophene	alliaceous	-	-	-	-	1	MS,RI,O
11	(*E*)-Methyl-1-propenyl disulfide	sauce	2	1	1	1	-	MS,RI,O
12	(*E*)-1-Propenyl propyl trisulfide	coconut	-	-	-	-	2	MS,RI,O
Nitrogen-containings								
1	2-Methylpyrazine	nutty	-	-	1	-	3	MS,RI,O
2	2,6-Dimethylpyrazine	roasted	-	-	-	-	3	MS,RI,O
3	2,3-Dimethylpyrazine	walnut	-	2	-	-	-	MS,RI,O
4	2-Ethyl-6-methylpyrazine	roasted potato	3	4	-	-	2	MS,RI,O
5	Ethenylpyrazine	nutty	-	1	-	-	-	MS,RI,O
6	3-Ethyl-2,5-dimethylpyrazine	potato	-	4	-	-	2	MS,RI,O
7	2-Methyl-3,5-diethylpyrazine	green grass	-	3	-	-	2	MS,RI,O
8	5,6,7,8-Tetrahydroquinoxaline	musty	-	3	-	-	1	MS,RI,O
9	2-Acetyl pyrrole	nutty	-	1	2	3	3	MS,RI,O
10	2-Methyl-5-propenylpyrazine	vanilla	-	2	-	-	-	MS,RI,O
Heterocyclics								
1	2-Furyl methyl ketone	sweet	3	1	4	3	2	MS,RI,O
2	5-Methyl furfural	maple	-	2	-	3	3	MS,RI,O
3	2(5H)-Furanone	buttery	4	-	-	1	-	MS,RI,O
4	5-Methyl-2-furfuryl alcohol	sweet	-	-	-	2	-	MS,RI,O
5	Furaneol	sweet	4	4	4	3	2	MS,RI,O
6	Furyl hydroxymethyl ketone	alcoholic	-	-	2	2	-	MS,RI,O
7	2-Furanmethanol	burnt	4	3	-	2	2	MS,RI,O
8	Dihydropyranone	sweet	3	-	-	-	-	MS,RI,O
9	2,3-Dihydro-3,5-dihydroxy-6-methyl-4-pyrone	warm	-	2	-	3	3	MS,RI,O
Unknown								
1	unknown	alcoholic	-	4	-	-	-	O
2	unknown	oil	-	-	-	4	-	O
3	unknown	vanilla	-	-	-	-	4	O
4	unknown	coconut	-	-	4	-	-	O

^a^ Aroma compounds detected in fried oils. ^b^ Odor description perceived by GC−O analysis. ^c^ Identification based on Nist 14 mass spectral database (MS); published RIs; published odor descriptions (O). ‘-’ not detected.

**Table A3:** Comparative analysis of the volatile compounds detected in five fresh Allium plants by gas chromatography-mass spectrometry (GC-MS).

No.	Compounds	RI ^a^ (Literature Value/Calculated)	Content (μg/g)				
			Green Onion	Garlic	Shallot	Onion	Bunching Onion
Alcohols							
1	Allyl alcohol	1122/1119	-	7.56 ± 1.86	-	-	-
2	2-Hexyl-1-decanol	2760/1347	3.2 ± 0.64	-	-	-	-
3	2-Ethylhexanol	1518/1515	-	13.13 ± 7.28	0.36 ± 0.2	-	2.74 ± 1.27
4	2-Ethyl-3-pentanol	1583/1583	-	-	-	-	3.51 ± 3.31
5	1-(2-Butoxyethoxy)ethanol	1800/1803	-	0.77 ± 0.27	-	-	11.51 ± 5.25
6	Phenylethyl Alcohol	1923/1924	-	-	0.86 ± 0.21	-	-
	Total		3.2	21.46	1.22	4.12	17.76
Aldehydes							
1	Hexanal	1097/1092	4.72 ± 0.83	0.2 ± 0.14	5.15 ± 1.44	4.29 ± 2.7	1.61 ± 1.37
2	2-Methyl-2-pentenal	1172/1172	14.16 ± 0.73	0.04 ± 0.01	11.69 ± 2.81	-	12.98 ± 6.51
3	2-Methylbutyraldehyde	1518/1485	-	-	-	13.93 ± 6.45	-
4	2-Undecenal	1755/1761	0.66 ± 0.31	-	-	-	-
5	2-Heptenal	1320/1320	-	-	0.65 ± 0.42	-	0.74 ± 0.24
6	Octanal	1300/1309	1 ± 0.41	-	-	-	-
	Total		20.54	0.24	17.49	18.22	15.33
Ketones							
1	Acetoin	1287/1300	-	0.58 ± 0.39	23.41 ± 2.4	-	10.34 ± 3.48
2	3-Hydroxy-3-methyl-2-butanone	-/1356	-	-	-	6.9 ± 3.14	-
3	3-methyl-2-Pentanone	1167/1356	-	0.13 ± 0.07	26.62 ± 5.73	-	-
4	2-Hydroxy-3-pentanone	1386/1385	-	-	-	-	2.64 ± 1.36
5	Tetrahydrocarvone	1552/1587	0.24 ± 0.04	-	-	-	-
6	3-Nonen-2-one	2073/2062	-	-	-	5.8 ± 3.48	-
7	1-(2-hydroxy-5-methylphenyl)ethanone	2178/2181	-	-	0.32 ± 0.02	-	-
	Total		0.24	0.71	50.35	12.7	12.98
Acids							
1	2-Propylpentanoic acid	1069/1061	-	-	-	0.85 ± 0.25	-
2	Hexanoic acid	1870/1873	5.27 ± 0.9	-	-	8.74 ± 3.28	2.29 ± 1.87
3	Nonanoic acid	2168/2167	3.94 ± 3.95	-	4.2 ± 1.4	7.87 ± 2.73	2.81 ± 1.25
4	Octanoic acid	2073/2068	2.48 ± 2.43	0.3 ± 0.24	3.71 ± 0.72	6.31 ± 0.98	3.71 ± 1.53
	Total		11.69	0.3	7.91	23.77	8.81
Esters							
1	Triacetin	2077/2075	1.41 ± 1.84	-	-	-	-
2	Methyl palmitate	2207/2191	-	-	0.68 ± 0.1	-	-
3	Propylene carbonate	1870/1850	0.28 ± 0.3	-	-	1.3 ± 1.54	5.57 ± 2.85
	Total		1.69	-	0.68	1.3	5.57
Nitrogen-containing compounds							
1	2-Methoxy-3-(1-methyl-propyl) pyrazine	1514/1519	-	-	0.17 ± 0.09	-	-
	Total		-	-	0.17	-	-
Sulfur-containing-compounds							
1	Dimethyl disulfide	1085/1078	-	3.65 ± 1.76	-	-	5.83 ± 1.63
2	3-Methylthiophene	1078/1129	-	-	0.29 ± 0.08	-	1.3 ± 0.23
3	Diallyl sulfide	1143/1152	-	3.14 ± 1.81	-	-	-
4	Propylallyl sulfide	1166/1166	-	0.09 ± 0.07	-	1.8 ± 1.16	-
5	S-Propyl thioacetate	1200/1197	2.07 ± 0.32	-	1.24 ± 0.41	-	1.44 ± 0.46
6	2,5-Dimethylthiophene	1190/1204	-	-	1.59 ± 0.27	-	-
7	Propanethial S-Oxide	-/1235	141.68 ± 30.12	-	130.82 ± 40.78	77.52 ± 39.85	108.25 ± 34.56
8	Methyl propyl disulfide	1242/1241	20.82 ± 4.27	0.2 ± 0.09	-	-	51.84 ± 13.33
9	3,4-Dimethylthiophene	1253/1263	43.14 ± 12.41	0.66 ± 0.45	-	-	58.34 ± 10.22
10	2,4-Dimethylthiophene	1253/1267	-	-	32.1 ± 10.04	-	-
11	Methyl allyl disulfide	1296/1294	-	18.85 ± 8.33	-	-	-
12	(*E*)-Methyl-1-propenyl disulfide	1300/1303	-	-	-	0.4 ± 0.32	-
13	Ethylisopropyl persulfide	1319/1311	-	-	-	0.21 ± 0.13	-
14	Propyl disulfide	1390/1391	192.32 ± 20.85	-	86.72 ± 76.87	24.97 ± 11.03	-
15	(*Z*)-propenyl propyl disulfide	1450/1426	-	-	-	-	19.8 ± 6.88
16	(*E*)-Propenyl propyl disulfide	-/1449	17.13 ± 1.94	0.19 ± 0	37.81 ± 20.78	7.57 ± 3.18	-
17	1-Allyl-2-isopropyldisulfane	-/1451	-	0.99 ± 0.4	-	-	59.17 ± 18.96
18	Diallyl disulfide	1475/1487	-	17.86 ± 10.92	-	-	-
19	(*E*)-1-Propenyl 2-propenyl disulfide	-/1500	-	14.67 ± 5.57	-	-	-
20	1,2-Dithiacyclopentene	1513/1526	-	7.17 ± 2.97	-	-	-
21	Ethyl 3-methylthiopropionate	1560/1555	0.37 ± 0.07	-	-	-	-
22	Dimethyl sulfoxide	1579/1575	1.39 ± 0.25	0.25 ± 0.15	-	-	2.88 ± 0.51
23	Methyl allyl trisulfide	1592/1589	-	5.47 ± 1.43	-	-	-
24	Dipropyl trisulfide	1672/1671	45.12 ± 4.65	-	43.42 ± 18.14	-	66.84 ± 11.67
25	3-Vinyl-1,2-dithiacyclohex-4-ene	-/1736	-	56.66 ± 42.25	-	-	-
26	1,4-Dimethyltetrasulfane	1742/1737	-	0.18 ± 0.04	-	-	-
27	1,2,5-Trithiepane	1781/1768	4.17 ± 0.28	-	-	4.28 ± 2.36	10.99 ± 6.23
28	Diallyl trisulfide	1787/1787	-	5.32 ± 1.71	-	-	-
29	(*E*)-1-Propenyl propyl trisulfide	1781/1786	8.59 ± 1.61	-	13.76 ± 4.71	30.67 ± 16.52	6.71 ± 2.28
30	1,3-Vinyldithin	1833/1830	-	50.95 ± 14.07	-	-	-
31	*(E*)-2-Ethyl-3-methylthiophane	-/1846	9.67 ± 1.04	-	10.42 ± 2.29	25.48 ± 8.68	13.35 ± 9.39
32	2,3-Dimercapto-1-propanol	-/1970	5.17 ± 3.06	-	3.44 ± 1.19	-	12.61 ± 3.14
33	1-(1-(Prop-1-en-1-ylthio)propyl)-2-propyldisulfane	-/2083	9.4 ± 1.05	-	16.67 ± 3.69	4.02 ± 1.31	5.73 ± 1.79
34	6-Ethyl-4,5,7,8-tetrathianonane	-/2136	1.81 ± 1.39	-	-	-	5.23 ± 1.29
35	(*E*)-3,6-Diethyl-1,2,4,5-tetrathietane	2168/2155	0.71 ± 0.21	-	2.28 ± 0.56	2.07 ± 0.92	-
36	2-Ethylthiophene	1173/1180	-	0.09 ± 0.03	-	-	-
37	(*E*, *E*)-Propenyl disulfide	1781/1760	-	4.2 ± 1.95	-	3.09 ± 1.66	-
38	Dimethyl trisulfide	1389/1392	-	1.97 ± 1.03	-	-	144.61 ± 50.89
39	Ethyl propyl sulfide	-/1713	0.97 ± 0.41	-	0.65 ± 0.57	-	0.81 ± 0.58
40	Methyl propyl trisulfide	1531/1533	-	-	0.37 ± 0.39	-	23.94 ± 8.12
	Total		504.53	192.56	381.58	182.08	599.67
Heterocyclics							
1	2-Hexyl-5-methyl-3(2H)-furanone	1999/1987	-	-	-	-	6.99 ± 2.73
2	Furaneol	2024/1964	1.11 ± 0.01	-	-	-	-
	Total		1.11	-	-	-	6.99

^a^ Ratio of calculated RI values to literature RI values on TG-WAX. ‘-’ not detected.

**Table A4:** Comparative analysis of the volatile compounds detected in five fried Allium oils by GC-MS.

No.	Compounds	RI ^a^ (Literature Value/Calculated)	Content (μg/g)				
			Green Onion-Oil	Garlic-Oil	Shallot-Oil	Onion-Oil	Bunching Onion-Oil
Alcohols							
1	Allyl alcohol	1122/1118	-	0.93 ± 0.83	-	-	-
2	Ethyl vinyl carbinol	1130/1130	2.16 ± 0.04	0.33 ± 0.3	3.53 ± 0.74	9.01 ± 1.28	-
3	1-Pentanol	1270/1266	7.08 ± 0.79	0.31 ± 0.28	10.03 ± 1.83	20.06 ± 3.24	-
4	Cyclopentanol	1339/1336	-	-	-	4.27 ± 0.87	-
5	1-Hexanol	1371/1371	-	0.05 ± 0.05	1.26 ± 0.16	-	-
6	4-Methyl-1-pentanol	1314/1315	0.94 ± 0.06	-	-	2.05 ± 0.4	-
7	1-Octen-3-ol	1476/1470	-	1.21 ± 1.06	17.28 ± 3.85	31.02 ± 4.8	5.62 ± 1.25
8	2-Ethylhexanol	1518/1515	-	-	1.95 ± 0.28	2.56 ± 0.32	-
9	4-Ethylcyclohexanol	-/1559	2.25 ± 0.48	0.24 ± 0.21	4.65 ± 0.88	8.22 ± 1.26	1.21 ± 0.25
10	1-Octanol	1573/1574	0.75 ± 0.17	0.02 ± 0.02	0.84 ± 0.06	0.96 ± 0.06	0.34 ± 0.08
11	(*E*)-2-octenol	1639/1635	-	0.08 ± 0.08	1.17 ± 0.45	-	-
12	Cyclooctyl alcohol	1640/1638	1.29 ± 0.43	-	-	-	0.31 ± 0.11
13	2-Dodecanol	1820/1667	-	-	0.36 ± 0.26	-	-
14	Decyl alcohol	1767/1762	-	-	-	-	0.59 ± 0.09
15	1-(2-Butoxyethoxy)ethanol	1800/1798	-	0.39 ± 0.34	-	-	-
16	Benzyl alcohol	1895/1886	2.16 ± 0.79	-	0.41 ± 0.11	-	0.66 ± 0.27
17	Phenylethyl Alcohol	1923/1923	0.11 ± 0.07	-	-	-	-
18	1-Dodecanol	1977/1976	-	-	4.37 ± 1.29	-	-
19	2-Octenol	1639/1636	-	-	-	2.38 ± 0.12	-
	Total		16.74	3.56	45.85	80.53	8.73
Aldehydes							
1	Hexanal	1097/1094	28.26 ± 0.63	1.73 ± 1.48	38.43 ± 4.23	67.72 ± 9.86	17.38 ± 1.45
2	2-Methyl-2-butenal	1093/1097	1.26 ± 0.09	0.22 ± 0.19	-	-	3.92 ± 0.51
3	(*E*)-2-Pentenal	1138/1136	1.5 ± 0.05	0.26 ± 0.22	2.84 ± 0.46	6.34 ± 1.4	0.89 ± 0.05
4	2-Methyl-2-pentenal	1172/1167	-	0.01 ± 0.01	0.07 ± 0.02	-	3.34 ± 0.26
5	Heptaldehyde	1195/1196	2.44 ± 0.19	0.15 ± 0.13	3.45 ± 0.58	5.95 ± 1.08	2.51 ± 0.1
6	(*E*)-2-Hexenal	1233/1220	0.23 ± 0.01	0.22 ± 0.19	-	-	1.76 ± 0.06
7	2-Hexenal	1216/1234	3.27 ± 0.09	-	3.57 ± 0.58	8.96 ± 1.42	-
8	Octanal	1300/1304	1.08 ± 0.22	0.16 ± 0.14	0.67 ± 0.18	-	-
9	(*E*)-2-Heptenal	1342/1342	-	-	-	-	60.09 ± 4.78
10	1-Nonanal	1400/1409	8.34 ± 2.02	0.6 ± 0.54	12.9 ± 3.5	-	5.7 ± 0.85
11	(*E*)-2-Octenal	1437/1442	8.98 ± 2.61	0.48 ± 0.43	15.12 ± 3.4	24.12 ± 4	4.33 ± 1
12	(*E*, *E*)-2,4-Heptadienal	1498/1482	25.82 ± 4.57	2.85 ± 2.47	49.26 ± 8.88	69.87 ± 10.62	23.98 ± 2.05
13	Benzaldehyde	1532/1530	-	0.08 ± 0.06	-	-	-
14	(*E*)-2-Nonenal	1549/1548	1.88 ± 0.59	-	2.38 ± 0.55	2.65 ± 0.46	-
15	p-Tolualdehyde	1638/1627	-	0.03 ± 0.02	-	-	-
16	(*E*)-2-Decenal	1660/1651	-	0.3 ± 0.28	9.34 ± 2.36	13.42 ± 0.1	-
17	Benzeneacetaldehyde	1661/1653	70.79 ± 16.37	0.34 ± 0.11	11.81 ± 4.24	7.03 ± 1.1	18.87 ± 5.57
18	(*E*, *E*)-2,4-Nonadienal	1708/1690	0.27 ± 0.09	-	0.48 ± 0.12	0.21 ± 0.02	-
19	2,4-Nonadienal	1668/1679	-	-		0.3 ± 0.02	-
20	2-Undecenal	1755/1757	1.44 ± 0.45	0.1 ± 0.06	1.95 ± 0.44	1.68 ± 0.06	-
21	(*E*, *E*)-2,4-Decadienal	1807/1819	23.51 ± 7.31	3.61 ± 1.29	82.45 ± 16.64	62.02 ± 5.41	12.43 ± 3.96
22	2-Phenyl-2-butenal	1907/1902	2.47 ± 0.34	0.04 ± 0.04	-	-	0.4 ± 0.15
23	2-Heptenal	1320/1320	65.71 ± 5.74	4.4 ± 3.74	78.09 ± 12.05	163.33 ± 32.44	-
	Total		247.25	15.58	312.81	433.6	155.6
Ketones							
1	3-Methyl-2-pentanone	1016/1016	-	-	2.59 ± 0.17	-	-
2	(*E*)-3-Penten-2-one	1121/1133	-	0.01 ± 0	-	-	-
3	2-Heptanone	1199/1193	0.73 ± 0.03	0.01 ± 0.01	0.51 ± 0.1	0.86 ± 0.16	-
4	3-Hydroxy-3-methyl-2-butanone	1247/1254	1.13 ± 0.04	0.06 ± 0.06	0.75 ± 0.1	1.23 ± 0.24	0.68 ± 0.09
5	Acetoin	1287/1299	0.91 ± 0.04		0.4 ± 0.09	0.42 ± 0.09	1.57 ± 0.28
6	Hydroxyacetone	1319/1312	17.06 ± 0.23	0.07 ± 0.06	7.24 ± 1.05	10.2 ± 1.29	21.52 ± 0.74
7	2,3-Octadione	1325/1323	17.49 ± 15.64	0.72 ± 0.62	-	39.64 ± 7.58	16.63 ± 2.56
8	2-Cyclopentenone	1349/1349	0.28 ± 0.09	-	0.12 ± 0.02	0.19 ± 0.03	-
9	3-Octen-2-one	1429/1425	-	-	-	0.6 ± 0.16	-
10	Acetoxy-2-propanone	1470/1469	1.36 ± 0.2	-	-	-	0.84 ± 0.15
11	2-Decanone	1508/1512	0.07 ± 0.03	-	-	-	-
12	3-Nonen-2-one	1518/1528	-	0.16 ± 0.15	3.88 ± 0.92	4.94 ± 0.68	-
13	(*E*)-3-Nonen-2-one	1523/1528	-	-	-	-	0.95 ± 0.28
14	3,5-Octadien-2-one	1531/1533	-	0.02 ± 0.02	-	-	-
15	(*E*, *E*)-3,5-Octadien-2-one	1585/1581	-	-	0.69 ± 0.16	0.89 ± 0.18	0.23 ± 0.04
16	4-Cyclopentene-1,3-dione	1576/1596	7.81 ± 0.68	-	2.26 ± 0.45	4.11 ± 0.77	-
17	2-Methyl-2-butenolide	1726/1722	-	-	-	-	0.77 ± 0.21
18	2(5H)-Furanone	1767/1751	4.38 ± 0.46	0.03 ± 0.03	1.6 ± 0.36	2.62 ± 0.61	0.73 ± 0.11
19	2-Hydroxy-2-cyclopenten-1-one	1769/1783	-	-	2.54 ± 1	3.24 ± 0.43	0.04 ± 0.02
20	Methyl cyclopentenolone	1829/1840	-	-	-	1.08 ± 0.12	-
21	2,3-Dihydro-3,5-dihydroxy-6-methyl-4-pyrone	2240/2214	-	0.64 ± 0.5	23.88 ± 8.96	48.81 ± 2.44	-
22	Dihydropyranone	1838/1850	0.43 ± 0.15	-	-	1.37 ± 0.06	0.27 ± 0.11
23	2,5-Octanedione	-/1350	-	-	19.6 ± 3.79	-	-
	Total		51.65	1.72	66.06	120.2	44.23
Acids							
1	Acetoxyacetic acid	-/1410	-	-	11.66 ± 2.97	0.12 ± 0.05	-
2	Acetic acid	1441/1474	24.15 ± 6.04	-	0.71 ± 0.12	9.13 ± 2.27	0.45 ± 0.09
3	Propionic acid	1564/1575	-	-	0.43 ± 0.06	1.31 ± 0.17	-
4	Pivalic acid	1579/1602	-	0.05 ± 0.04	0.3 ± 0.27	-	-
5	Isovaleric acid	1671/1698	1.56 ± 0.62	-	-	-	-
6	Pentanoic acid	1750/1772	-	-	-	-	0.41 ± 0.17
7	Hexanoic acid	1870/1864	8.87 ± 1.2	0.27 ± 0.04	10.66 ± 0.58	10.73 ± 2.13	5.01 ± 1.06
8	Octanoic acid	2073/2063	3.36 ± 0.86	0.05 ± 0.05	2.2 ± 0.67	1.04 ± 0.15	3.07 ± 2
9	Nonanoic acid	2168/2158	7.24 ± 1.13	-	2.31 ± 0.87	-	6.16 ± 4.76
	Total		49.2	0.37	31.52	22.33	15.1
Esters							
1	4-Hydroxybutyric acid	-/1631	4.6 ± 0.7	-	3.1 ± 0.81	2.15 ± 0.43	-
2	2-Methylpentanoic acid methyl ester	-/1723	-	-	1.4 ± 0.36	1.58 ± 0.05	-
3	Tetrahydro-2-pyranone	1785/1796	1.24 ± 0.32	-	-	-	-
4	Acetic acid 2,3-dihydroxypropyl	-/1941	-	-	1.55 ± 0.28	0.84 ± 0.24	-
	Total		5.84	-	6.05	4.57	-
Lactones							
1	Butyrolactone	1635/1633	-	-	-	-	4.48 ± 0.46
2	5-Methyl-3-methylenedihydrofuran-2(3H)-one	-/1703	1.47 ± 1.24	0.18 ± 0.16	6.25 ± 1.47	-	-
	Total		1.47	0.18	6.25	-	4.48
Sulfur-containing compounds							
1	Dimethyl disulfide	1085/1071	19.76 ± 1.02	1.15 ± 1.01	2.47 ± 0.53	2.49 ± 0.43	20.7 ± 0.7
2	3-Methylthiophene	1078/1128	0.24 ± 0.06	-	0.13 ± 0.07	0.14 ± 0.03	0.86 ± 0.03
3	2-Methylthiophene	1093/1093	-	0.03 ± 0.03	-	0.14 ± 0.02	-
4	Diallyl sulfide	1143/1152	-	0.31 ± 0.28	-	-	-
5	Methyl propyl disulfide	1242/1246	6.53 ± 0.25	-	3.27 ± 0.56	1.44 ± 0.32	9.34 ± 0.92
6	3,4-Dimethylthiophene	1253/1263	5.51 ± 0.29	0.03 ± 0.03	3.32 ± 0.68	13.76 ± 2.58	8.57 ± 0.94
7	Methyl allyl disulfide	1296/1290	-	3.97 ± 3.37	-	-	-
8	Dimethyl trisulfide	1389/1381	74.7 ± 4.31	3.48 ± 2.98	22.63 ± 3.25	29.02 ± 5.12	76.44 ± 6.14
9	Propyl disulfide	1390/1391	1.71 ± 0.47	-	6.44 ± 1.53	7.36 ± 1.61	1.67 ± 0.17
10	(*E*)-Propenyl propyl disulfide	-/1449	2.78 ± 2.54	-	6.59 ± 1.46	4.19 ± 0.7	6.65 ± 1.42
11	Diallyl disulfide	1475/1489	-	6.32 ± 5.54	-	-	-
12	Methyl propyl trisulfide	1531/1528	11.42 ± 2.5	0.01 ± 0.01	9.73 ± 2.06	3.31 ± 0.53	18.78 ± 4.33
13	Dimethyl sulfoxide	1579/1572	3.27 ± 0.54	0.04 ± 0.03	1.5 ± 0.43	-	2.36 ± 0.95
14	Methyl allyl trisulfide	1592/1589	-	9.33 ± 8.37	-	-	-
15	(*E*)-1-Methyl propenyl trisulfide	-/1605	5.42 ± 1.93	0.03 ± 0.03	1.98 ± 0.74	3.85 ± 0.75	7.52 ± 3.15
16	Dipropyl trisulfide	1672/1670	-	-	-	-	2.33 ± 0.79
17	3-Thiophenecarboxaldehyde	1693/1695	-	0.21 ± 0.14	-	-	-
18	Ethyl propyl sulfide	-/1713	0.64 ± 0.63	-	-	0.24 ± 0.04	0.73 ± 0.17
19	5-Methylthiophene-2-carboxaldehyde	1735/1734	-	0.03 ± 0.02	-	-	-
20	(*Z*)-1-Allyl-3-propenyl trisulfane	-/1769	-	-	0.96 ± 0.23	2.19 ± 0.25	-
21	Diallyl trisulfide	1787/1782	-	6.75 ± 1.73	-	-	-
22	(*E*)-1-Propenyl propyl trisulfide	1781/1786	-	-	-	-	0.73 ± 0.21
23	Ethyl 2-hydroxyethyl sulfide	-/1846	8.88 ± 1.52	-	4.52 ± 0.99	-	0.92 ± 0.19
24	Dimethyl sulfone	1912/1909	-	-	-	-	0.93 ± 0.2
25	2-Methyl-2-thiazoline	1303/1307	-	0.01 ± 0.01	-	-	-
26	Allyl isothiocyanate	1369/1375	-	0.02 ± 0.02	-	0.06 ± 0.03	-
27	Ethyl propyl sulphide	1038/1041	-	-	1.33 ± 0.25	4.74 ± 0.66	-
28	Trimethyl thiazole	1398/1404	-	0.03 ± 0.03	-	-	-
	Total		140.86	31.75	64.87	72.93	158.53
Nitrogen-containing-compounds							
1	Pyridine	1188/1190	-	0.04 ± 0.04	-	-	-
2	2-Methylpyrazine	1271/1275	-	0.53 ± 0.45	-	-	25.02 ± 1.82
3	2,5-Dimethyl pyrazine	1333/1332	-	0.99 ± 0.85	2.83 ± 0.58	-	60.89 ± 5.54
4	2,6-Dimethylpyrazine	1334/1334	1.81 ± 0.15	-	-	-	-
5	Ethylpyrazine	1344/1348	-	-	0.07 ± 0.01	-	1.72 ± 0.04
6	2,3-Dimethylpyrazine	1345/1356	-	0.03 ± 0.02	-	-	3.18 ± 0.07
7	2,3-Dimethylpyridine	1358/1367	-	-	-	-	0.16 ± 0.06
8	3-Ethylpyridine	1387/1385	-	0.1 ± 0.09	-	-	-
9	2-Ethyl-6-methylpyrazine	1398/1394	0.37 ± 0.04	1.09 ± 0.93	0.33 ± 0.04	-	9.5 ± 1.42
10	Trimethyl-pyrazine	1416/1416	-	-	-	-	6.76 ± 1.64
11	1,2,4-Triazole	-/1420	-	-	6.04 ± 1.08	3.11 ± 0.41	-
12	5-Ethyl-2-methylpyridine	1413/1429	-	0.04 ± 0.05	-	-	-
13	3-Ethyl-2,5-diMethylpyrazine	1459/1454	0.94 ± 1.24	1.79 ± 1.58	0.26 ± 0.07	-	17.96 ± 4.79
14	2-Vinyl-6-methylpyrazine	1491/1497	-	0.3 ± 0.26	-	-	-
15	2,3-Diethyl-5-methylpyrazine	1499/1502	-	0.04 ± 0.03	-	-	-
16	2-Methyl-3,5-diethylpyrazine	1505/1506	-	-	-	-	0.26 ± 0.04
17	2,5-Dimethyl-3-propylpyrazine	1525/1526	-	-	-	-	2.13 ± 0.75
18	2-Methyl-5-propenylpyrazine	1535/1546	-	0.35 ± 0.32	-	-	6.24 ± 1.66
19	N-Acetyl-2-methyl-1-propaneamine	1731/1749	-	-	0.11 ± 0.02	-	0.04 ± 0.02
20	Acetamide	1763/1763	-	-	0.21 ± 0.12	-	1.71 ± 0.18
21	3-(Methylthio)pyridine	1791/1791	-	0.04 ± 0.04	-	-	-
22	Quinoxaline	1905/1899	0.1 ± 0.05	-	-	-	0.12 ± 0.04
23	Acrylamide	1943/1942	6.76 ± 1.75	-	3.84 ± 0.85	-	8.84 ± 1.65
24	2-Acetyl pyrrole	1971/1968	32.45 ± 10.07	0.25 ± 0.08	13.74 ± 3.11	20.08 ± 0.87	13.8 ± 3.88
25	Pyrrole-2-carboxaldehyde	2019/2015	-	0.12 ± 0.03	2.66 ± 0.41	1.84 ± 0.22	-
26	2-Pyrrolidinone	2002/2021	-	0.01 ± 0.02	-	-	2.69 ± 0.93
27	2-Piperidinone	2060/2085	-	-	-	-	1.08 ± 0.36
28	1-(6-Methyl-2-pyrazinyl)-1-ethanone	1688/1710	-	-	-	-	1.1 ± 0.41
29	2-Formyl-3,4-dihydro-2H-pyran	-/1716	1.3 ± 1.14	-	-	0.93 ± 0.1	0.27 ± 0.12
	Total		43.73	5.72	30.09	25.96	163.47
Heterocyclics							
1	2-Butylfuran	1123/1147	0.12 ± 0.01	-	-	-	-
2	2-Pentylfuran	1258/1252	3.7 ± 0.48	0.79 ± 0.7	3.93 ± 0.9	4.11 ± 0.76	4.9 ± 0.87
3	2-Furyl methyl ketone	1515/1520	16.73 ± 1.67		4.21 ± 1.22	8.14 ± 1.58	2.9 ± 0.32
4	5-Methyl furfural	1570/1582	34.16 ± 4.63	0.17 ± 0.16	7.2 ± 1.33	16.63 ± 3.16	1.4 ± 0.33
5	5-Methyl-2-furfuryl alcohol	1729/1743	4.1 ± 0.89	-	2.15 ± 0.43	0.6 ± 0.17	1.35 ± 0.06
6	2,5-Furandicarboxaldehyde	1969/1965	1.05 ± 0.32	-	-	0.61 ± 0.03	-
7	Furyl hydroxymethyl ketone	2001/1989	26.36 ± 7.05	0.05 ± 0.05	6.12 ± 1.48	7.74 ± 0.72	1.14 ± 0.29
8	Furaneol	2037/2029	2.54 ± 1.08	0.08 ± 0.08	1.73 ± 0.58	0.98 ± 0.29	4.33 ± 1.14
9	5-Acetoxymethyl-2-furaldehyde	2194/2171	5.31 ± 0.43	0.05 ± 0.05	-	0.9 ± 0.14	-
10	2-Hexyl-5-methyl-3(2H)-furanone	1999/1987	-	-	-	-	1.66 ± 0.52
11	2-Furanmethanol	1686/1674	33.76 ± 3.03	0.31 ± 0.27	15.89 ± 2.59	20.03 ± 3.25	4.33 ± 0.38
12	Furfural	1474/1481	114.73 ± 7.41	-	-	121.48 ± 17.01	-
13	2-Furanacrolein	1851/1859	-	0.01 ± 0.01	-	-	-
	Total		242.56	1.46	41.23	181.22	22.01

^a^ Ratio of calculated RI values to literature RI values on TG-WAX. ‘-’ not detected.

**Table A5:** The number of volatile compounds in fresh Allium plants and their fried oils identified in GC-MS analysis.

	Fresh Allium Plants	Fried Allium Oils
Sulfur-containing compounds	44	32
Nitrogen-containing compounds	1	31
Ketones	7	23
Aldehydes	6	23
Alcohols	7	19
Heterocyclics	2	13
Acids	4	11
Esters	4	5
Lactones	0	2
